# Poison severity score and sequential organ failure assessment score: Carbon monoxide poisoning prognosis

**DOI:** 10.1371/journal.pone.0212025

**Published:** 2019-03-01

**Authors:** Il Jae Wang, Seok-Ran Yeom, Sung-Wook Park, Sung-Hwa Lee, Sang-Kyoon Han, Soon-Chang Park, Ji-Ho Ryu, Seong-Youn Hwang

**Affiliations:** 1 Department of Emergency Medicine, Biomedical Research Institute, Pusan National University Hospital, Busan, South Korea; 2 Department of Emergency Medicine, Pusan National University Yangsan Hospital, Gyeongsangnam-do, Yangsan, South Korea; 3 Department of Emergency Medicine, Samsung Changwon Hospital, Gyeongsangnam-do, Changwon, South Korea; Azienda Ospedaliero Universitaria Careggi, ITALY

## Abstract

**Purpose:**

We aimed to examine the utility of the Poison Severity Score (PSS) and Sequential Organ Failure Assessment (SOFA) score as early prognostic predictors of short-term outcomes in patients with carbon monoxide (CO) poisoning. We hypothesized that both the PSS and the SOFA score would be useful prognostic tools.

**Methods:**

This was retrospective observational study of patients with CO poisoning who presented to the emergency department and were admitted for more than 24 hours. We calculated PSS, the initial SOFA score, a second (2^nd^) SOFA score, and a 24-hour delta SOFA score. The primary outcome was reported as the cerebral performance category (CPC) scale score at discharge. We classified those with CPC 1–2 as the good outcome group and those with CPC 3–5 as the poor outcome group.

**Results:**

This study included 192 patients: 174 (90.6%) belonged to the good outcome group, whereas 18 (9.4%) belonged to the poor outcome group. The PSS (1.00 [0.00, 1.00] vs 3.00 [3.00, 3.00], p < 0.001), initial SOFA (1.00 [0.00, 2.00] vs 4.00 [3.25, 6.00], p < 0.001), 2^nd^ SOFA score (0.00 [0.00, 1.00] vs 4.00 [3.00, 7.00], p < 0.001), and 24-hour delta SOFA score (-1.00 [-1.00, 0.00] vs 0.00 [-1.00, 1.00], p = 0.047) of the good outcome group were significantly higher than those of the poor outcome group. The areas under the receiver operating characteristic curve for PSS and the initial SOFA and 2^nd^ SOFA scores were 0.977 (95% confidence interval [CI] 0.944–0.993), 0.945 (95% CI 0.903–0.973), and 0.978 (95% CI 0.947–0.994), respectively.

**Conclusion:**

The PSS, initial SOFA score, and 2^nd^ SOFA score predict acute poor outcome accurately in patients with CO poisoning.

## Introduction

Carbon monoxide (CO) is produced during incomplete combustion of carbon containing compounds; it is a colorless, odorless, tasteless, non-irritant gas [1]. CO exists in a very low concentration (less than 0.001%) in the atmosphere, and a small amount exists naturally in the human body [2, 3]. However, exposure of an individual to high concentrations of CO can be fatal [4]. CO toxicity is estimated to account for more than half of the fatal poisonings in many countries [5]. It has a high prevalence rate globally, and in the United States, more than 50,000 patients visit emergency departments annually because of CO poisoning [6, 7]. In Korea, unintentional CO poisoning is decreasing, but the rate of suicide by CO poisoning is steadily increasing, and it is now the fourth most common suicide method [8].

CO has a 200-fold higher affinity for hemoglobin than does oxygen. Therefore, even at low concentrations, carboxyhemoglobin (COHb) is formed rapidly and the oxygen dissociation curve is shifted to the left [4]. This reduces oxygen transport to tissues and vital organs with high oxygen demand, such as the brain and heart, which are generally the most damaged [9]. Persistent comas, delayed neurologic sequelae, and myocardial infarction can occur [1]. In addition, CO can damage various parts of the body as it can cause rhabdomyolysis, acute kidney injury, pancreatitis, and pulmonary edema [5].

Many prognostic factors have been studied to predict the prognosis of CO poisoning. However, the majority of studies have identified the severity of patients on the basis of clinical symptoms, shock, receipt of hyperbaric oxygen therapy, presence of identifiable cardiac markers, and delayed neurologic sequelae [10–14]. Very few studies have applied a scoring system, which comprehensively evaluates various clinical features and laboratory tests, to evaluate CO poisoning.

The Poison Severity Score (PSS) is a scoring system that grades the severity of poisoning according to clinical symptoms [15]. PSS has been used to evaluate a variety of different poisonings, but reports on PSS for assessing the prognosis of CO poisoning are rare [16–18]. A Sequential Organ Failure Assessment (SOFA) score assesses the severity of a patient's disease by evaluating six organ systems [19]. However, there are no studies using SOFA scores in patients with CO poisoning.

The aim of this study was to evaluate the utility of PSS and serial SOFA scores as early prognostic predictors of short term outcomes in patients with CO poisoning. We hypothesize that both the PSS and the serial SOFA score would be very useful prognostic tools.

## Methods

This study was approved by the Institutional Review Board of Pusan National University Hospital. (1805-003-066). Informed consent was waived because the data were analyzed anonymously and retrospectively. The emergency department (ED)was located in a single, urban, tertiary-care hospital that has more than 30,000 annual visits and more than 800 poisoning cases each year.

### Study design and setting

This was a retrospective observational study of patients with CO poisoning who presented to the emergency department of our hospital in Korea between January 2014 and March 2016. Data were extracted retrospectively from the hospital electronic medical records.

The diagnosis of CO poisoning was based on carboxyhemoglobin levels >2% in non-smokers and >9% in smokers. For patients who were transferred from another hospital, CO poisoning was diagnosed on the basis of the carboxyhemoglobin levels obtained at the previous hospital.

The exclusion criteria were as follows: 1) age < 15 years; 2) those with cirrhosis, chronic kidney failure, and other underlying diseases that may affect the SOFA score; 3) those who co-ingested other drugs except alcohol; 4) those who were discharged, died, or transferred within 24 hours because serial SOFA could not be measured. All patients who were diagnosed with CO poisoning and treated with normobaric 100% oxygen therapy or hyperbaric oxygen therapy (HBOT) were included. The criteria for determining whether a patient should undergo HBOT are not yet clear [4]. In addition, patients who could potentially benefit from HBOT have not yet been determined [20,21]. As the carboxyhemoglobin level at presentation is likely to be underestimated because of the passage of time or delivery of oxygen during transport, if the patient or family agrees [4], the treatment guideline at our hospital recommends all patients with CO poisoning be treated with HBOT, specifically three times in 12-hour intervals. Patients who were intubated were not treated with hyperbaric oxygen therapy.

### Data collection

We investigated the demographic data (age, sex), source of CO, intentionality, carboxyhemoglobin (COHb) level, arterial blood pH, lactate levels, transfer status (from another hospital or not), initial symptom(s), mortality, HBOT and Cerebral Performance Categories (CPC) scale at discharge. We also calculated the PSS and serial SOFA score. The PSS and initial SOFA score were calculated on the basis of the patient’s condition at the time of arrival to the emergency department. The 2^nd^ SOFA score was calculated after 24 hours of ED arrival. The 24-hour delta SOFA score (defined as the 2^nd^ SOFA score minus the initial SOFA score) was then calculated. One of the authors randomly selected 20% of the patients and measured their CPC scale score and PSS. There was no interobserver disagreement. The primary outcome was the CPC scale score at discharge [22]. We classified those with a CPC 1–2 as the good outcome group and those with a CPC 3–5 as the poor outcome group.

### Statistical analysis

For the continuous variables, normality was determined by a Shapiro-Wilks test. Continuous variables with normal distribution were expressed as mean and standard deviation. Continuous variables without normal distribution were expressed as median and interquartile range. Continuous variables were analyzed using a two-sample t-test or Wilcoxon rank-sum test. Categorical variables were expressed as numbers of cases and percentages and evaluated using Fisher's exact test. A receiver operating characteristic (ROC) analysis was performed to identify the prognostic utility of PSS and serial SOFA scores for the patients with CO poisoning. Sensitivity, specificity, positive predictive value, and negative predictive value were presented as the cut-off value using the Youden index with the highest sensitivity and specificity. All analyses were performed using MedCalc 18, R 3.3. A p-value less than 0.05 was considered statistically significant.

## Results

### Patient selection and characteristics

**Fig 1:**
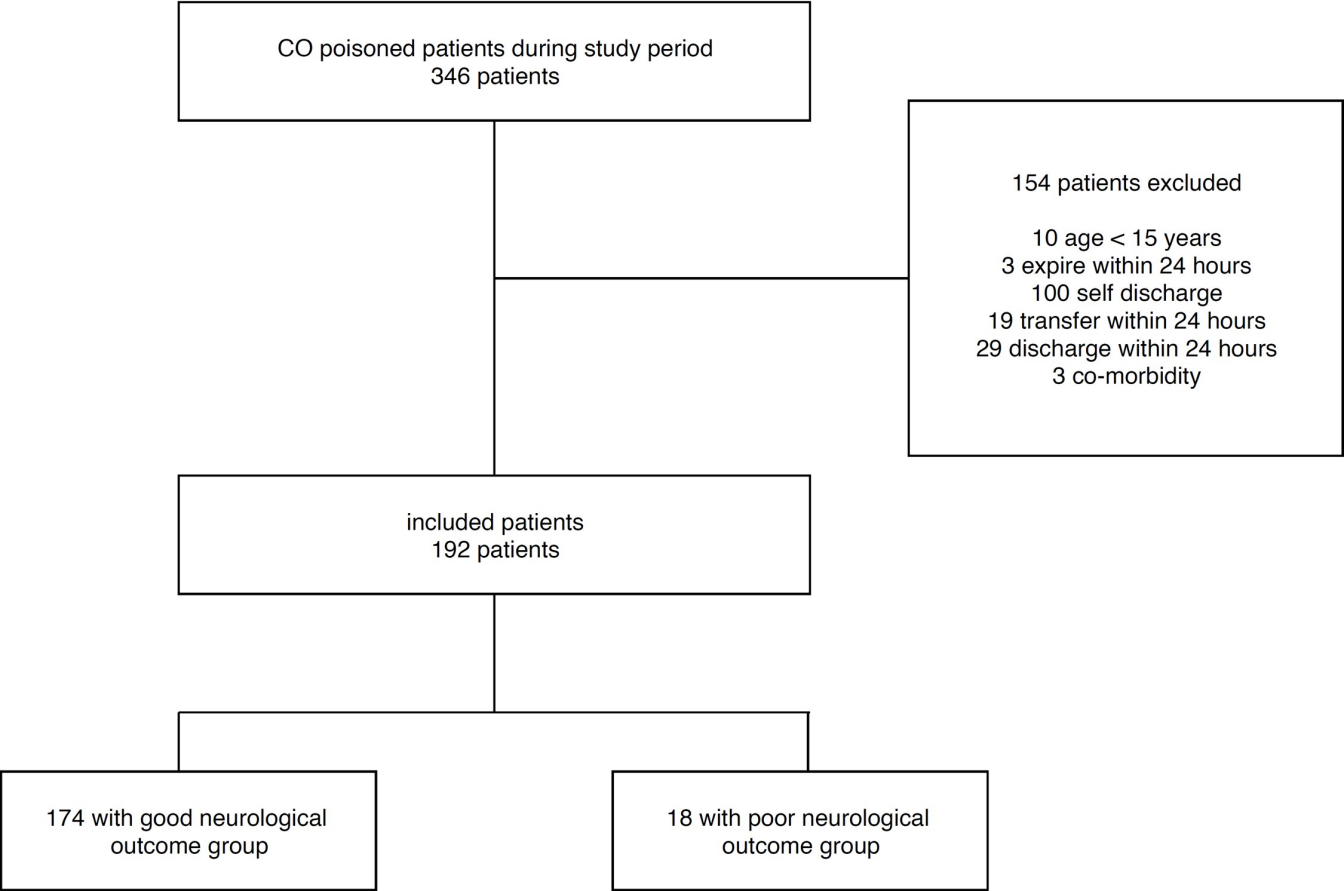
Study flow diagram of CO poisoned patients. CO: Carbon monoxide.

Between January 2014 and March 2016, a total of 347 consecutive patients were identified with acute CO poisoning. Of these patients, 154 patients were excluded. Finally, 192 patients were included in this study(Fig 1). The patients included 140 male (72.9%) and 52 female (26.8%) patients. The mean age was 46.52 ± 16.84 years. The most common source of CO was ignition coal and the second most common was from briquettes. The CO poisoning was intentional in 139 (72.4%) patients and the number of patients who came from other hospitals was 71 (37.0%). The most common initial symptom was decreased mental function (51.6%) and headache (14.6%). Other symptoms were chest discomfort (n = 6), dyspnea (n = 2), nausea (n = 4), sore throat (n = 4), dizziness (n = 5), and abdominal pain (n = 2). The median level of COHb was 15.70 [5.85, 29.27] and the median level of lactic acid was 2.85 [1.58, 6.00]. Three patients died during hospitalization. The median PSS, initial SOFA score, 2^nd^ SOFA score, and 24-hour delta SOFA score were 1.00 [0.00, 1.00], 1.00 [0.00, 2.25], 0.00 [0.00, 1.00], and -1.00 [-1.00, 0.00], respectively. The baseline characteristics of the patients are summarized in Table 1.

**Table 1:** General characteristics according to clinical outcome at discharge.

	Total (n = 192)	Poor outcome group (n = 18; 9.4%)	Good outcome group (n = 174; 64.4%)	p
Age (y), mean ± SD	46.52 ± 16.84	54.50 ± 15.32	45.69±16.81	0.032
Sex, n (%)				0.268
Female	52 (27.1)	7 (38.9)	45 (25.9)	
Male	140 (72.9)	11 (61.1)	129 (74.1)	
Transfer from other hospital, n (%)				0.609
yes	71 (37.0)	8 (44.4)	63 (36.2)	
no	121 (63.0)	10 (55.6)	111 (63.8)	
Intentional exposure, n(%)				<0.001
yes	139 (72.4)	9 (50.0)	130 (74.7)	
no	41 (21.4)	1 (5.6)	40 (23.0)	
unknown	12 (6.2)	8 (44.4)	4 (2.3)	
Source of carbon monoxide, n(%)				0.802
briquette	45 (23.4)	4 (22.2)	41 (23.6)	
scene of fire	17 (8.9)	2 (11.1)	15 (8.6)	
ignite charcoal	106 (55.2)	9 (50.0)	97 (55.7)	
the others	24 (12.5)	3 (16.7)	21 (12.1)	
Initial symptoms, n(%)				<0.001
normal	42 (21.9)	0 (0.0)	42 (24.1)	
altered mental	99 (51.6)	18 (100.0)	81 (46.6)	
headache	28 (14.6)	0 (0.0)	28 (16.1)	
the others	23 (12.0)	0 (0.0)	23 (13.2)	
Lactic acid (mmol/L), median [IQR]	2.85 [1.58, 6.00]	3.95 [2.92, 8.55]	2.70 [1.50, 5.38]	0.020
pH, median [IQR]	7.41 [7.36, 7.44]	7.38 [7.37, 7.45]	7.41 [7.36, 7.44]	0.605
COHb, median [IQR]	15.70 [5.85, 29.27]	15.45 [1.47, 42.50]	15.70 [6.20, 29.17]	0.963
PSS, median [IQR]	1.00 [0.00, 1.00]	3.00 [3.00, 3.00]	1.00 [0.00, 1.00]	<0.001
GCS, median [IQR]	14.00 [11.00, 15.00]	6.00 [5.25, 7.00]	15.00 [13.00, 15.00]	<0.001
Initial SOFA, median [IQR]	1.00 [0.00, 2.25]	4.00 [3.25, 6.00]	1.00 [0.00, 2.00]	<0.001
2nd SOFA, median [IQR]	0.00 [0.00, 1.00]	4.00 [3.00, 7.00]	0.00 [0.00, 1.00]	<0.001
24-hour delta SOFA, median [IQR]	-1.00 [-1.00, 0.00]	0.00 [-1.00, 1.00]	-1.00 [-1.00, 0.00]	0.047
CPC, n(%)				
1	169 (88.0)	0 (0.0)	169 (97.1)	
2	5 (2.6)	0 (0.0)	5 (2.9)	
3	3 (1.6)	3 (16.7)	0 (0.0)	
4	12 (6.2)	12 (66.7)	0 (0.0)	
5	3 (1.6)	3 (16.7)	0 (0.0)	
Death, n	3	3	0	

CPC, cerebral performance category; COHb, carboxyhemoglobin; PSS, poisoning severity score; SOFA, sequential organ failure assessment

### Unadjusted analysis

For our analysis, we compared the good outcome group with the poor outcome group. The good outcome group consisted of 174 (90.6%) patients and poor outcome group consisted of 18 patients (9.4%). Age was significantly higher in the poor outcome group than in the good outcome group. The PSS (1.00 [0.00, 1.00] vs 3.00 [3.00, 3.00]), initial SOFA (1.00 [0.00, 2.00] vs 4.00 [3.25, 6.00]), 2^nd^ SOFA (0.00 [0.00, 1.00] vs 4.00 [3.00, 7.00]) and 24-hour delta SOFA (-1.00 [-1.00, 0.00] vs 0.00 [-1.00, 1.00]) of the good outcome group were also significantly higher than those of the poor outcome group. The lactate levels were also statistically higher in the poor outcome group. There was no significant difference with respect to the sex (p = 0.265) and carboxyhemoglobin levels (p = 0.531) between the two groups.

### ROC analysis

**Fig 2:**
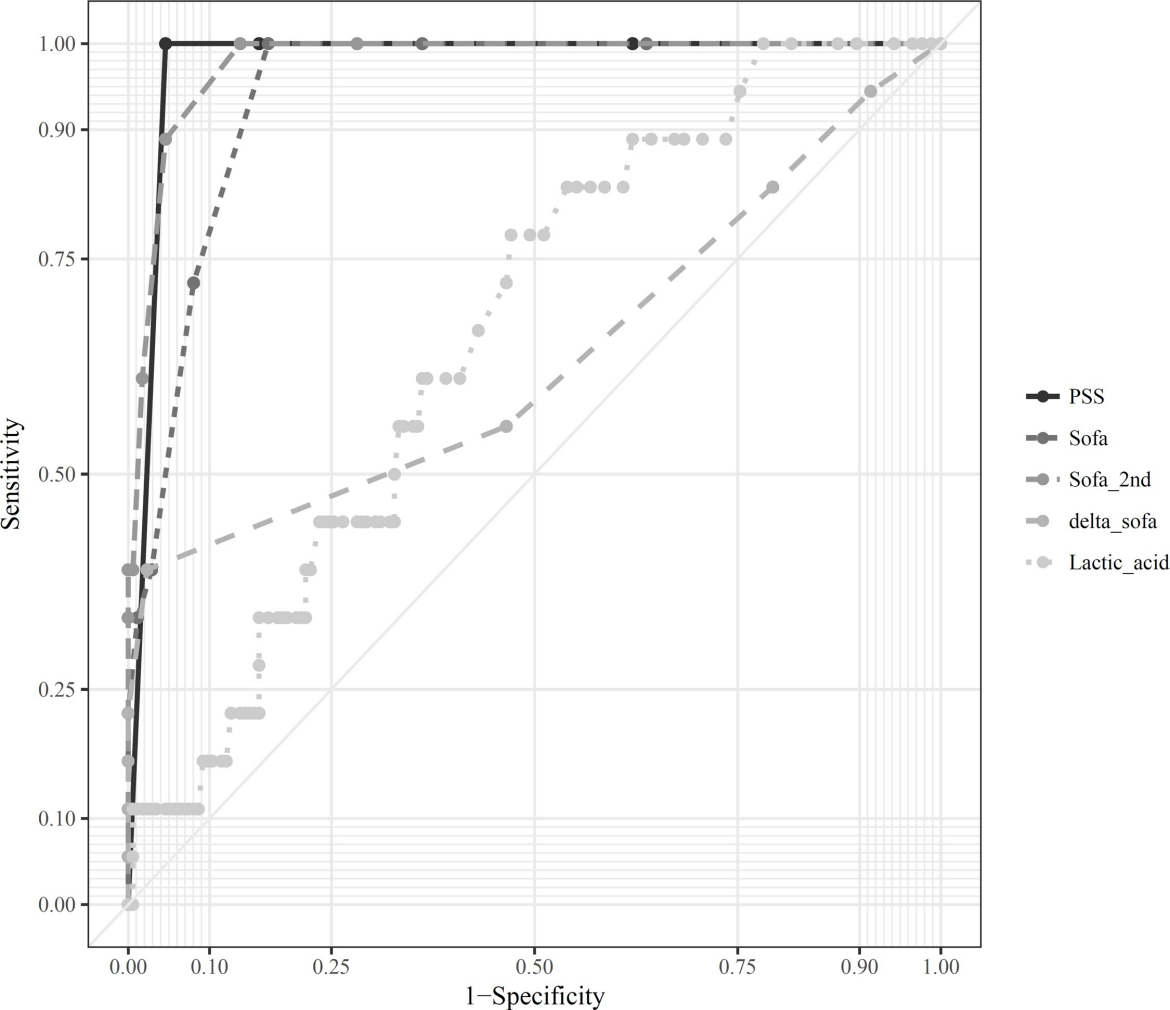
Receiver operating characteristic curve of PSS, SOFA, 2^nd^ SOFA, delta SOFA, lactic acid to predict the poor outcome. PSS, poisoning severity score; SOFA, sequential organ failure assessment.

An ROC curve analysis was performed for PSS, initial SOFA score, 2^nd^ SOFA score, 24-hour delta SOFA score, and lactic acid levels (Fig 2). The AUC for the PSS, initial SOFA score, and 2^nd^ SOFA score was 0.977, 0.945, and 0.978, respectively, and they showed a very high predictive power. The AUC of the 24-hour delta SOFA score and lactate levels was 0.635 and 0.667, respectively. Table 2 shows the optimal cut off value, sensitivity, specificity, positive predictive value, and negative predictive value, (Table 2).

**Table 2:** Prognostic accuracy table.

AUC (95% CI)	Cutoff¶	Sensitivity (95% CI)	Specificity (95% CI)	PPV (95% CI)	NPV (95% CI)	+LR (95% CI)	-LR (95% CI)
0.977 (0.944–0.993)	>2	100 (81.5–100.0)	95.4 (91.1–98.0)	69.2 (53.3–81.6)	100.0	21.8 (11.1–42.8)	0.0
0.945 (0.903–0.973)	>2	100 (81.5–100.0)	82.76 (76.3–88.1)	37.5 (30.2–45.4)	100.0	5.8 (4.2–8.0)	0.0
0.978 (0.947–0.994)	>1	100 (81.5–100.0)	86.21 (80.2–91.0)	42.9 (34.1–52.1)	100.0	7.3 (5.0–10.5)	0.0
0.635 (0.562–0.703)	>0	38.89 (17.3–64.3)	97.7 (94.2–99.4)	63.6 (36.1–84.4)	93.9 (91.4–95.7)	16.9 (5.5–52.3)	0.6 (0.4–0.9)
0.667 (0.595–0.733)	>2.8	77.78 (52.4–93.6)	52.87 (45.2–60.5)	14.6 (11.3–18.6)	95.8 (90.6–98.2)	1.7 (1.2–2.2)	0.4 (0.2–1.0)

¶ The “optimal” cutoff value was defined by the highest Youden index value (sensitivity + specificity −1)

PSS, poisoning severity score; SOFA, sequential organ failure assessment; AUC, area under curve; CI, confidence interval, PPV, positive predictive value; NPV, negative predictive value; LR, likelihood ratio

## Discussion

We investigated the accuracy of PSS and serial SOFA scores for predicting acute poor outcomes in patients with CO poisoning. The PSS, initial SOFA score, and 2^nd^ SOFA scores were able to predict acute poor outcomes accurately in the patients. When the cutoff value was >2, the AUC of PSS was 0.977. In addition, when the cutoff values of the initial SOFA and the 2^nd^ SOFA were > 2, and > 1, the AUC values were 0.945 and 0.978, respectively. The delta SOFA score and lactic acid level showed a statistically significant correlation with the acute prognosis of CO poisoning, but the AUC value was relatively low.

There have been many studies on the prognosis of CO in relation to laboratory test results such as lactic acid levels, COHb levels, and cardiac enzymes [14, 23, 24]. Very few studies have applied the scoring system to patients with CO poisoning. In this study, PSS and SOFA scores were applied to patients with CO poisoning and both showed good predictive power.

The PSS was developed in the 1990s by three organizations: the European Association of Poisons Centers and Clinical Toxicologists (EAPCCT), the International Programme on Chemical Safety (IPCS), and the European Commission. PSS is a scoring system that evaluates the severity of poisoned patients based on 12 clinical categories. The overall score is based on sub-scores of the most serious categories. The PSS has been used to assess patients exposed to organophosphate pesticides, and carbamate pesticides, but it is not yet clear whether the PSS can be used for evaluating patients with CO poisoning [16, 17]. The study by Cevic et al. in 2006 is the only report to assess the utility of the PSS for patients with CO poisoning [18]. These researchers reported that a total of 182 patients with CO poisoning, all of whom either died or experienced serious medical complications, had a grade 2 PSS or higher. Similar results were obtained in our study. All patients with poor outcome had a grade 3 PSS.

The SOFA score, another scoring system we applied in this study, assessed the severity of the patient’s status by evaluating six organ systems in the intensive care unit (ICU). The SOFA score is an objective score based on neurological status and laboratory results. To the best of my knowledge, no studies have applied the SOFA score to evaluate patients with CO poisoning. The results of this study show that the SOFA score has a very high predictive power. CO is mainly toxic to the heart and brain but it is also toxic to the whole body and therefore the SOFA score, which evaluates multiple organs simultaneously, is thought to be useful [25]. We expected the delta SOFA score to be highly predictive, but it had a poor predictive power compared with the PSS and the initial and 2^nd^ sofa scores. This is presumably because of the relatively low number of patients in the poor outcome group. There were 4 patients with a delta SOFA score of 3 or more and all of them were in the poor outcome group.

The relationship between lactic acid levels and CO has been assessed in many studies. Dogan et al. stated that lactate levels were high in the group requiring HBO and Cervellin et al. reported that initial blood lactate levels correlated with clinical severity [26, 27]. Moon et al. also suggest that the initial lactate levels were an independent factor associated with complications [28]. However, in our study, the predictive power of lactate was lower than that of the SOFA score or PSS; the PSS and SOFA scores are a comprehensive scoring system in which various parameters are considered, whereas the lactate level is a single laboratory test.

Patients with CO poisoning, like patients with cardiac arrest, may not be able to recover consciousness if they experience severe brain damage and this is very important for both doctors and patients’ families to understand. The PSS and SOFA score can be helpful for understanding this problem as both scores predict acute outcomes very accurately.

Our study has some limitations. First, as a retrospective study, we were unable to collect information objectively and relied on medical chart reviews. Second, this is a single center study, so it is difficult to generalize. Third, we could not perform hyperbaric oxygen therapy for patients requiring intubation as our facility’s HBO chamber does not support mechanical ventilation. Fourth, we did not follow up with patients after discharge, so we could not get information about the occurrence of delayed neurologic sequelae.

## Conclusions

We show that PSS and SOFA scores can accurately predict the prognosis of patients with CO poisoning. Both PSS and SOFA scores had a higher predictive power than lactic acid levels, which is normally used in CO poisoning. These scores can be useful for doctors in predicting the prognosis of CO poisoning and for the patients’ families in understanding the potential outcome.
